# Some Views on the Present Crisis

**Published:** 1919-12-27

**Authors:** 


### THE RECONSTRUCTION OF CHARITY.

### Some Views on the Present Crisis.

It is symptomatic of the confusion of the present time that the land of England has been changing hands on a scale which can only be compared to the upheaval which followed the dissolution of the monasteries. One of the first results of that earlier change was the destruction of the ecclesiastical system of charity, and it is not surprising that this destruction was shortly followed by the passing of the first Poor Law in the reign of Queen Elizabeth. The old personal tie between the corporate landlord of the monastic house and its dependants was rudely broken. The new men endowed with church lands regarded little but their own enrichment. Charity, as hitherto understood, was abolished. The State stepped in, and the child of State intervention was the much abused Poor Law. Such in outline was the history of charity on the previous occasion when the land of England passed from its traditional to a new set of proprietors. Has it no lessons for us when a similar revolution has occurred? The parallel is curious, and shows that there is no empty rhetoric in the phrase which heads this article.

### The Inevitable.

The reconstruction of charity, then, is inevitable, because it cannot be dissociated from the revolution which has occurred among large and small proprietors of land. There would not have been this change if riches generally had not been redistributed. Mushroom fortunes have been made. Some millionaires have grown richer. The middle-classes with small private properties have largely been improverished. Tradesmen have become rich. The result is that the public with the means to support charitable institutions is not the same public as of old, and, what is more serious, that the new possessors of wealth have not the tradition nor the habit of giving. There is no more common mistake than to suppose that any man of sense can spend a large income suddenly acquired sensibly. The truth is that the qualities which helped him to acquire it are hindrances to its wise expenditure. If the making of money deserves to be called an art, the spending of money is certainly an art also. It is difficult to acquire this art late in life. As the old proverb has it, it takes three generations to make a gentleman: for a new family is like a new house. It has first to be founded, then to weather, then to be civilised by time.

### A Vicious Circle.

Hospitals therefore know hardly where to look for support at the moment when they most need it, for the needs of the war have created an active demand for beds which before the war was largely ignored. The consequence is that the hospitals are turning to the State, the activities of which received a new impetus because of that vast organisation which it was compelled to improvise during the recent struggle. At the same time the State also is at its wits' end for money, and the vicious circle seems to be complete.

The only way to alleviate the situation is to begin to educate the newly enriched classes, and it remains to be seen whether they can be educated in time to prevent the preponderating share of hospital finance from being borne by the State. It is easier to state the difficulty than to solve it. For it will hardly be denied that the principal sums recently given to hospitals have come from those who have increased already existing fortunes. The new prosperous classes are as sheep without a shepherd. The sensation of an easy flow of coin is still so strange that they vacillate between an itch to spend and an ache to hoard it. Of the former many queer stories could be told. The salesmen who on demobilisation have returned to their shops confess with disgust or amusement that they must unlearn their old technique of persuasion. The customers who now come to them are of a new type, often with no idea but to purchase the most expensive article. It will take time to civilise such people. Like gamblers they clutch at their gains, and would gape at the suggestion that money can only give dignity or decency to those who care how it is spent, and that generosity, until it is an instinctive habit of the mind, is a mood built on the sand at the mercy of every gust of pique or passion.

### The Working-man's Subscription.

Thus we find that the hospitals in the great industrial centres, largely supported by working men's subscriptions, are often regarded as the property of their purse-proud supporters. They say that they have a right of entry to them, when they should say that they have given in their hour of health willing aid to those others who are ill, and in whose company mischance or accident may some time throw them.

### The Habit of Giving.

But if giving is a habit difficult for the uncivilised to gain, it is not quickly lost, and those who are less able to give than they were can do excellent work by serving on hospital committees and subsidiary organisations to leaven the newcomers with the traditional spirit which they represent. If this leavening is not maintained during the next quarter of a century, history will repeat itself. The long arm of the State will be invoked as it was in the days of Elizabeth, and under whatever name it may be called a new State system of relief alarmingly like the old Poor Law will be created. Our one hope lies in the fact that the voluntary hospitals, unlike the monasteries, have survived the change and are there to humanise it. That is the final reason why it is now or never that we must rally to their support.

